# Supplementary material for: Oleic Acid-Containing Phosphatidylinositol Is a Blood Biomarker Candidate for SPG28
Source: Biomedicines. 2023 Apr 4;11(4):1092. doi: 10.3390/biomedicines11041092 (PMC10136216; doi:10.3390/biomedicines11041092)
Supplement: Supplementary file 1 [file biomedicines-11-01092-s001.zip › Supplementary Figure S1.pdf]

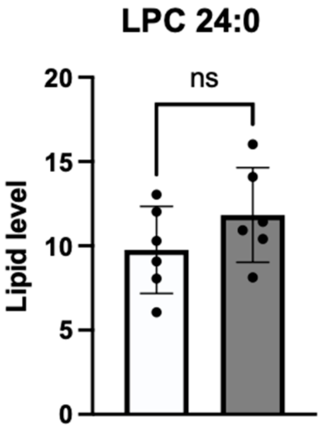

Figure S1. LPC 24:0 in plasma from mice. *Ddhd1*(+/+) mice and *Ddhd1*(-/-) mice are shown in opened and filled columns, respectively. The unit of the vertical axis is pmol/mg. Error bars indicate mean ±SD. ns: not significant. All data were analyzed by Student’s t-test.

Figure S1
